# Eye Troubles in Pregnancy

**Published:** 1892-06

**Authors:** 


### EYE TROUBLES IN PREGNANCY.

As the conclusion from a very interesting paper on the "Induction of Premature Labor, in Amaurosis and Amblyopia in Pregnancy" (*Jour. Am. Med. Asso.*), Dr. Pooley says:

1. In all cases of pregnancy, not only should examinations of the urine be systematically made, but the eyes should be examined with the ophthalmoscope; since, in a large proportion of cases where eye troubles exist, the patients make no complaint of disorders of vision. Frequently such troubles can be detected with the opthalmoscope long before any disease of the kidney is shown in the urine.

2. In uremic amaurosis, without changes in the eye visible to the ophthalmoscope, even should the usual accompanying symptoms, such as dizziness, nausea and threatened convulsions be absent, their supervention is soon to be anticipated, and the immediate induction of premature labor is indicated, without waiting until the life, as well as the sight of the patient is in danger.

3. In neuro-retinitis, the induction of premature labor is not only justifiable, but urgently demanded. In some instances it is called for even in the earlier months of pregnancy.

4. It is required in cases of eye trouble recurring in successive pregnancies.

5. A woman having suffered in this way during pregnancy, the relationship of cause and effect should be fully explained, both to herself and her husband.—*Med. Brief.*

